# A web-based resource for designing therapeutics against Ebola Virus

**DOI:** 10.1038/srep24782

**Published:** 2016-04-26

**Authors:** Sandeep Kumar Dhanda, Kumardeep Chaudhary, Sudheer Gupta, Samir Kumar Brahmachari, Gajendra P. S. Raghava

**Affiliations:** 1 Bioinformatics Centre, CSIR-Institute of Microbial Technology, Sector 39A, Chandigarh, India; 2 CSIR-Institute of Genomics and Integrative Biology, Mathura Road, New Delhi, India

## Abstract

In this study, we describe a web-based resource, developed for assisting the scientific community in designing an effective therapeutics against the Ebola virus. Firstly, we predicted and identified experimentally validated epitopes in each of the antigens/proteins of the five known ebolaviruses. Secondly, we generated all the possible overlapping 9mer peptides from the proteins of ebolaviruses. Thirdly, conserved peptides across all the five ebolaviruses (four human pathogenic species) with no identical sequence in the human proteome, based on 1000 Genomes project, were identified. Finally, we identified peptide or epitope-based vaccine candidates that could activate both the B- and T-cell arms of the immune system. In addition, we also identified efficacious siRNAs against the mRNA transcriptome (absent in human transcriptome) of all the five ebolaviruses. It was observed that three species can potentially be targeted by a single siRNA (19mer) and 75 siRNAs can potentially target at least two species. A web server, EbolaVCR, has been developed that incorporates all the above information and useful computational tools (http://crdd.osdd.net/oscadd/ebola/).

The Ebola virus (EBOV), came into the picture with the outbreak in 1976 in Sudan and subsequently in Zaire, where it was initially detected near the Ebola River (Zaire species) from which it received its current name^1^. It can infect both animals and humans. The medium of transmission of this virus is mainly body fluids such as milk, saliva, semen, urine, etc. The Ebola Virus Disease (EVD) is characterized by abrupt fever, stomach pain, headache, vomiting, and diarrhea^2^. Because the EVD has overlapping symptoms with other diseases such as malaria, flu and typhoid, it makes it all the more complicated to diagnose the infection from the symptoms^3^. The onset of the disease is confirmed using ELISA, PCR and virus isolation test^4^.

Once the disease has been diagnosed, the next challenge is the treatment and tackling symptoms by providing intravenous fluids for maintaining electrolytes, oxygen status and blood pressure^5^. Even so, there are limited options to treat this disease directly. Recently, ZMapp was invented against the Ebola virus (EBOV), which is a combination of 3 monoclonal antibodies^6^,^7^. Although it has not been tested in humans, successful animal studies have been carried out^8^. To the best of the authors’ knowledge, there is no known approved vaccine against this disease available at the moment^9^.

However, there are two promising vaccine candidates that are currently in phase-I of clinical trials^10^. The first candidate (ChAd3-ZEBOV) has been developed by GlaxoSmithKline (GSK) and the National Institute of Allergy and Infectious Diseases (NIAID)^11^. This vaccine comprises a glycoprotein gene from EBOV and Sudan Ebola Virus (SUDV) under the control of chimpanzee adenovirus 3. The second potential vaccine is rVSV-ZEBOV, developed by NewLink Genetics and Merck Vaccines USA in collaboration with the Public Health Agency of Canada that uses attenuated virus with glycoprotein of EBOV^12^. The rVSV-ZEBOV was found to be safe and effective in clinical trial results^13^.

In the past, RNAi has been proven to be a promising tool for the treatment of many viral diseases^14^ and has helped in circumventing viral infections^15^. A siRNA targeting the Zaire Ebola virus RNA polymerase L protein formulated in stable nucleic acid-lipid particles (SNALPs) showed complete protection in guinea pigs^16^. In a separate study, the authors have used a combination of siRNAs targeting L, VP24 and VP35 formulated in SNALPs and observed protection in two-third of the rhesus monkeys and in all macaques who were given seven post exposure treatments^17^. TKM-100802 is a set of small RNA molecules encapsulated in lipid nanoparticles that target the vital viral proteins (VP24, VP35, and L) and cleave viral RNA in the cells and prevent its multiplication^16^,^17^. A recent study shows that short interfering RNAs (siRNAs) packaged into lipid nanoparticles are able to protect all the rhesus monkeys under experimental design against Makona strain outbreak of Ebola virus^18^.

TKM-Ebola-Guinea drug trial is an outcome of the collaborative efforts of Tekmira (a Canadian company) and Oxford University; unfortunately, the clinical trial was halted recently as therapeutic benefits to the patients were not observed after reaching the predefined endpoint^19^.

Hence, there is a need to design computer-aided subunit vaccines and siRNA-based therapy against EVD. Fortunately, the genomes of the five ebolaviruses have been sequenced and annotated^20^,^21^. Thus, it is possible to develop computational approaches for designing potential therapy against the Ebola virus. In this study, we aim to create a web-based resource that provides information required for developing effective drugs or vaccines against this virus.

## Methods

### Sequence of Genomes

We downloaded the protein, and mRNA sequences of five ebolaviruses: Bundibugyo ebolavirus (BDBV) (Accession Number FJ217161), Reston ebolavirus (RESTV) strain Pennsylvania (Accession number AF522874), Sudan ebolavirus (SUDV) strain Gulu (Accession Number AF086833), Tai Forest ebolavirus (TAFV) (Accession Number FJ217162), and Ebola virus - Mayinga, Zaire, 1976 (EBOV-May) (Accession Number AF086833) from the NCBI^22^,^23^. These genomes have sizes between 18–19 kb with 40–42% GC content. There are seven genes present in EBOV with two variants of glycoproteins (*GP*) gene in RESTV and SUDV, while three splice variants have been annotated in other species. Following are the splice variants of GP; i) GP is the spike protein, ii) sGP: (soluble GP) is the default 7A variant and iii) ssGP: (small soluble GP) is a -1A(6A) variant. The alignment file for each protein/mRNA across the different species was generated using ClustalW^24^.

### Viral-specific conserved patterns

In order to investigate virus-specific conserved patterns, overlapping patterns of 9 amino acids (9mer) were generated, from proteins of above-mentioned five ebolaviruses. There were 43 proteins in total (including different splice variants of *GP* gene) from the five ebolaviruses. Further, all the possible 9mer peptides from each protein were predicted for their immunological potential. One of the challenges in vaccine development is to avoid self-tolerance as the body’s immune system rarely acts against self-antigens. Thus, all those 9mers that are available in the human body (or human genome) were removed. In order to achieve this goal, we removed all those viral patterns that are 100% identical to any pattern found in human thousand proteomes. Human thousand proteomes were created by using 1000 Genomes data and translating it to proteins sequences^25^,^26^. This led us to the 9mer peptide/pattern that is found in this virus but absent in human proteomes. In order to develop an effective vaccine against a wide range of species, it is important to identify conserved patterns across the different species of Ebola. Therefore, this study involves the identification of unique patterns of 9mer peptides along with the frequency of their occurrence across different ebolaviruses. Also, a high priority was given to a pattern, which was common in the most number of ebolaviruses, which led to patterns/peptides of size of 9 residues that were conserved among ebolaviruses.

### Pipeline for epitope prediction

After the exploration of 9mer peptides which were found to be conserved in different ebolaviruses but absent in human proteomes, our next goal was to identify peptides that could activate the human immune system in order to generate memory cells against Ebola virus. In order to understand the immunomodulatory or stimulatory effect of these peptides, we computed different types of epitopes as described below:

#### B-cell epitope prediction

In this study, we have used the recently developed method LBtope^27^ for predicting linear B-cell epitopes among the aforementioned conserved patterns. LBtope is a highly accurate method trained on a large dataset that contains experimentally validated B-cell epitopes and non-epitopes. We applied the LBtope model with default threshold of 60% probability cut-off. Similarly, conformational B-cell epitopes have been predicted in the above conserved peptides using CBTOPE^28^. CBTOPE is a unique method that predicts conformational B-cell epitopes in an antigen from its amino acid sequence as opposed to previous methods, which required the structure of the antigen to predict the same. The CBTOPE results were obtained on the default threshold of −0.3 cut-off and implemented on our website.

#### MHC alleles binding peptides

The major histocompatibility complex (MHC) is a set of cell surface molecules that plays a vital role in the human immune system. The major role of the MHC is to bind to antigenic regions (or peptides) and present these peptides on the cell surface, where appropriate T-cells recognize these peptides. Thus, it is important to predict MHC binders, as these binders have the ability to activate the T-cells of the immune system. In this study, we have used ProPred1 with top 4% cutoff (default) for predicting MHC Class I binders^29^. ProPred1 is a matrix-based program, which predicts the peptide binding potential for 47 MHC Class I alleles. These binders may activate cytotoxic T-lymphocyte (CTL), so we may call these binders as potential CTL epitopes, which were predicted using CTLPred with default parameters. Similarly, to predict potential T-helper epitopes, we predicted MHC Class II binders in the above conserved peptides. The prediction of MHC Class II binders was performed using ProPred^30^ with top 3% threshold as suggested by the authors of ProPred. It is a quantitative/virtual matrix-based method that allows the users to predict promiscuous MHC Class II binders for 51 alleles.

#### T-cell response prediction

In addition to the prediction of MHC binders which are potential T-cell epitopes, we also predicted CTL epitopes using a direct method, CTLPred^31^. CTLPred is a direct method that predicts T-cell epitopes (CTL) from a primary sequence of the antigen instead of using the intermediate step where MHC Class I binders are predicted. CTLPred has a number of modules (e.g., ANN, SVM) for predicting CTL epitopes; in this study we have used SVM-based module with default parameters. In the past, numerous methods have been developed for predicting MHC Class II binders but none of them has been able to predict the type of interleukins released by these binders. In this study, we have used a method, IFNepitope with a default threshold of 0.0 to predict the antigenic region or MHC binders that can activate Th1 cells (T-helper cell type I)^32^ responsible for releasing interferon-gamma (IFN-γ). In brief, we predicted IFN-γ inducing peptides in Ebola antigens using IFNepitope. Similarly, we also predicted antigenic regions that can activate Th2 cells responsible for releasing of cytokine, interleukin-4 (IL4). In this study, we used IL4pred, at a default threshold of 0.2, for predicting the IL4-inducing antigenic regions or peptides in Ebola antigens^33^.

### RNA therapeutics

In order to design siRNA-based therapeutics against the Ebola virus, we generated 19mer oligonucleotide from the corresponding mRNA sequences, as this length is sufficient to silence the cognate mRNA target sequence. In literature, 19mer is considered the core length of siRNA duplex (apart from 2 nucleotide overhang) that is used for RNAi-based therapy to silence a target transcript. Most of the prediction methods are developed based on 19mer length of siRNA sequence^34^,^35^,^36^.

**Figure 1:**
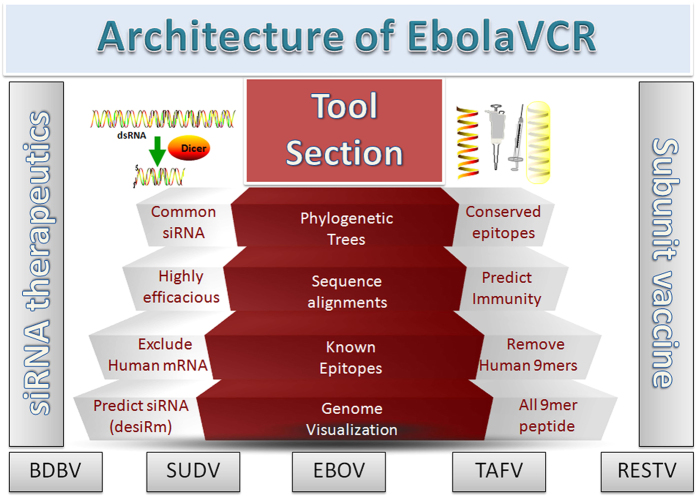
Overall architecture of EbolaVCR. Figures inside this figure are created with the help of ScienceSlides software (http://www.visiscience.com/).

First we generated 19mer overlapping oligonucleotides in each gene of the above-mentioned five ebolaviruses. Secondly, we removed all the oligonucleotides present in human genes sequenced so far, using human reference mRNA. Thirdly, we identified oligonucleotides conserved in different species of the virus. Finally, we got 19mer oligonucleotides conserved in the viral species but absent in the human genome. In order to identify which oligonucleotide could serve as an efficient siRNA against the Ebola virus, we predicted the efficacy of each oligonucleotide (siRNA) using DesiRm software with default parameters^34^. The final set of 19mers was further subgrouped gene-wise and in each subgroup, we separated the highly potent siRNAs (having efficacy greater than 0.80 value). The overall architecture of the EbolaVCR developed in this study has been shown in Fig. 1.

### Tool section

#### Phylogenetic trees

MEGA software version 5.0^37^ has been used for creating the phylogenetic tree from protein sequences of Ebola virus. In order to generate multiple sequence alignment of proteins we used the software ClustalW^24^. These alignments were used to construct the tree using Maximum-Likelihood algorithm where branch lengths were visualized from the given options.

#### Protein structure of Ebola antigens

In order to understand the function of Ebola proteins, we have predicted the tertiary structure of Ebola proteins. To assist the scientific community, we predicted tertiary structures of viral proteins using I-TASSER suite^38^. The templates for structure prediction were derived using Hhbuilt suite with default parameters. All the predicted protein structures for 38 proteins (except for L protein) are available from our web resource. Further, visualization tools have been integrated to visualize these structures. In addition to predicted structures, we have also provided the structure of Ebola virus proteins present in protein data bank (PDB). We believe these structures will be helpful in computer-aided drug design.

## Results

### Subunit vaccine against Ebola virus

#### 9mer peptides

In our study, we have generated all the possible overlapping 9mer peptides for each of the ebolavirus. We have removed the redundant as well as peptides found in human reference proteome (Table 1). Further, the peptides present in 1000 Genomes-based human proteome were also filtered to avoid any cross presentation with any individual. We have observed 2 peptides from RESTV and single peptide from SUDV, which were shared with human reference proteome. In addition to that, we have also observed a peptide from EBOV that was not present in the human reference proteome but present among the peptides of 1000 Genomes-based human proteome. The protein-wise distribution of peptides from different ebolaviruses is shown in supplementary files (Figure S1).

#### Vaccine candidates

**Figure 2:**
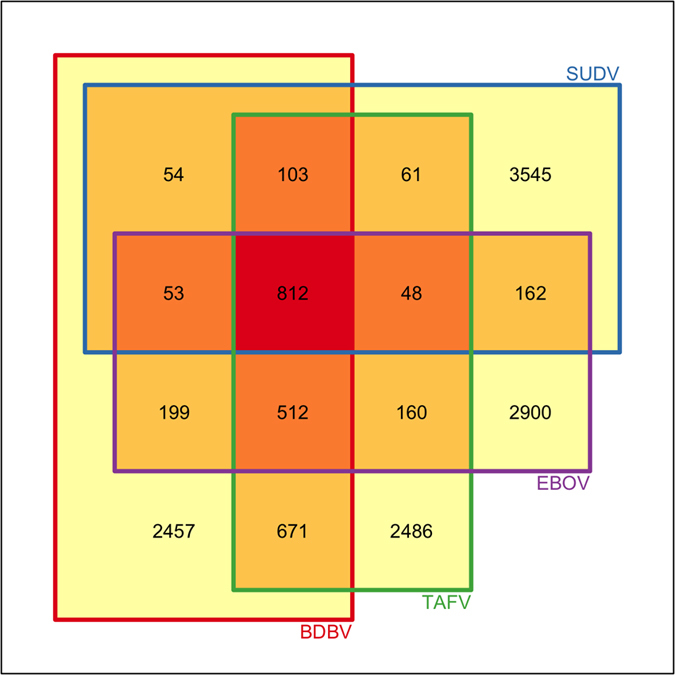
The Venn Diagram for five sets of peptides from four human pathogenic ebolaviruses.

The conservation in a peptide among the different ebolaviruses is helpful for its applicability in a broad spectrum. Therefore, we have extracted conserved peptides (9mer), and found that 629 peptides were shared among all the five ebolaviruses in our study (Figure S2) and 812 peptides were conserved among four human pathogenic ebolaviruses (excluding RESTV) (Fig. 2, Table 2). These common peptides, shared among different ebolaviruses, have the potential to design vaccine for all types of ebolaviruses. We computed protein-wise distribution of conserved peptides among different species of Ebola virus (Figure S3, Table S1). In order to identify conserved regions, we computed multiple sequence alignment of each protein of virus using ClustalW (Figures S4 and S5). We also predicted immunogenicity of these peptides, particularly of conserved peptides, using our immuno-pipeline. In our analysis, we have not found any of the conserved peptides with B-cell epitopes and promiscuous MHC binders. There were 21 peptides that were common among all the four human pathogenic ebolaviruses (excluding RESTV, not pathogenic to human). These peptides are highly promiscuous vaccine candidates (Table 2). We further analyzed 14 peptides that can activate most of the arms of the immune system (Table 3). Out of these 14 peptides, 2 were conserved in three species and three peptides were conserved in at least two ebolaviruses.

### siRNA-based therapy

#### Alignment of mRNA sequences

We performed the multiple sequence alignment (MSA) for genes across all the five ebolaviruses in order to identify the conserved regions in different genes. We were unable to locate a single 19mer oligonucleotide, common in all the five ebolaviruses. We got a stretch of maximum length of nine nucleotides across five ebolaviruses. In this study we used ClustalW software^24^ for MSA and Jalview^39^ visualization of alignment (Figure S6).

#### Potential siRNA suppressors

**Figure 3:**
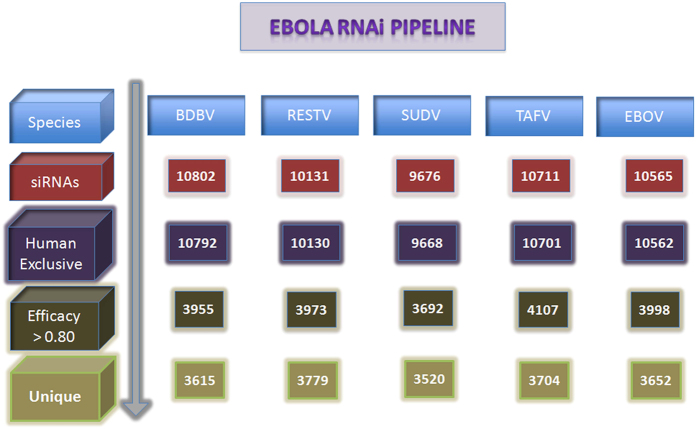
RNAi pipeline of EbolaVCR and number of potential therapeutic siRNA predicted for each ebolavirus.

**Figure 4:**
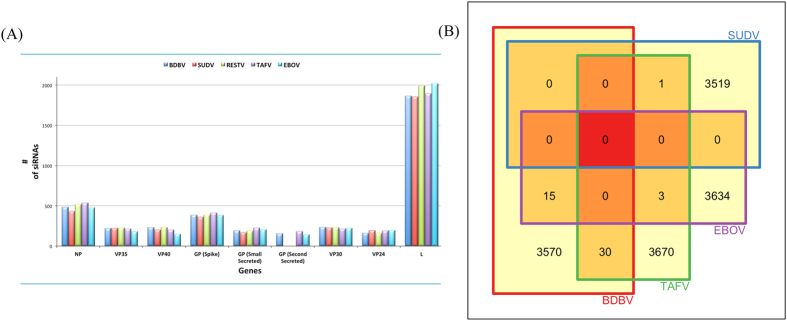
(**A**) Gene-wise distribution of siRNA across five ebolaviruses. (**B**) Shared siRNA across four human pathogenic ebolaviruses.

The specific 19mer oligonucleotides were generated in all the five ebolaviruses. Figure 3 shows the architecture of the Ebola RNAi pipeline, which presents the strategy adopted to identify potential therapeutic siRNAs. First, we computed the efficacy of each 19mer oligonucleotide using desiRm software at default threshold value of 0. Next, we identified 19mers, which were present in human genome in order to eliminate off-target interaction. Finally, we selected all those siRNAs, which may silence targets gene with efficacy more than a value of 0.80. We wanted to identify promiscuous siRNA that could target all five ebolaviruses. Unfortunately, we did not find a single common 19mer oligonucleotide in any of the five ebolaviruses (Figure S7). The distribution of siRNAs selected against Ebola across the five ebolaviruses at gene level has been shown in Fig. 4. The distribution of potential siRNAs against each target gene in ebolaviruses is shown in Table 4 and the number of siRNAs targeting common ebolaviruses is enlisted in Supplementary Table S2. Our server shows potential siRNAs against target genes in the form of tables with the Circos plots^40^ for visualization. Users can get detailed information like strain ID, gene, siRNA sequence, cognate mRNA sequence, starting position and efficacy from our website. Users can sort the table according to a position or efficacy to get the most efficacious siRNA along with other details. The detailed gene-wise distribution of siRNA from different ebolaviruses is shown in supplementary files (Figure S8).

### Integration of Computational Tools

In this web-based platform, we have integrated a number of computational tools to provide a comprehensive service to the scientific community working on Ebola virus. These tools will be very useful in designing therapeutics against new ebolavirus or drug resistant ebolavirus. Following is the brief description of modules integrated in EbolaVCR.

#### Mapping of experimentally validated epitopes

The Immune Epitope Database (IEDB) is the largest database of experimentally characterized antigenic regions or epitopes that may modulate immune system. In summary, IEDB maintains experimentally validated epitopes. In order to identify antigenic regions that have already been characterised in the past, we mapped experimentally proven IEDB epitopes on Ebola antigens. Here, we have extracted the epitopes using the filter of source organism as ebolavirus or name of any ebolavirus. We have linked these epitopes and their source organism(s) to the original source page of IEDB. The source molecule of these epitopes is linked to NCBI for detailed description. We have also provided the facility to map the peptides from the user sequence to IEDB, MHCBN and BCIPEP databases^41^,^42^,^43^.

#### Phylogenetic trees

It is important to understand the similarity among ebolaviruses or evolution of a species. In order to understand evolution in ebolavirus, we generated phylogenetic trees, based on similarity in their protein sequences. In this study, MEGA software has been used for generating phylogenetic trees using default parameters^37^. The trees are available at our web resource. In our analysis of phylogenetic trees for each gene, we have observed the evolution of ebolaviruses. We found that RESTV and SUDV have been coevolved from the same phylogenetic node, and BDBV and TAFV are closely related, while EBOV evolved from a distant node of ancestry.

## Discussion

### Promising vaccine epitopes

The vaccine against the Ebola virus is the need of the hour^6^,^9^,^10^. In this study, we made an attempt to analyze the conserved epitopes so that the resulting vaccine covers a broad range of species, sequenced till date. Besides conservation, immune response is the next challenge to be addressed by the vaccine candidates. We have predicted diverse kinds of immune responses for better efficacy of the candidates. In the past, computational studies have been carried out for designing vaccines against viruses^44^,^45^. But these studies were limited in terms of the number of proteins covered and the types of immune responses considered^46^,^47^,^48^. We have observed the peptides (9mers) that are capable of stimulating different kinds of immunity. In the current study, we have uncovered 123 conserved peptides among all the human pathogenic ebolavirus that can activate cytotoxic T lymphocytes (CTL). In addition to CTL activation, we have also identified 42 peptides that can activate the T helper response after binding with MHC class II. We have also eliminated the peptides that could share sequence identity with the human proteome to avoid any cross-reaction of vaccine candidates with human proteins. We are hopeful that these vaccine candidates will provide better immunity and lead to a successful vaccine in the future. We have also discovered 45 conserved peptides that were able to trigger B-cell response to generate antibody among all the human pathogenic subtypes of the Ebola virus. These peptides could serve in the diagnosis of the Ebola virus.

We have also examined experimentally validated epitopes available in the Immune Epitope Database (IEDB). It was observed that 1138 epitopes, covered in our study, out of 1513 peptides were available in IEDB. This demonstrates the validity of our predictions. In addition, we suggested 16 epitopes in our study, one of which has been validated experimentally to bind with 2 MHC alleles (HLA*24-02, HLA-B*07:02). The peptide (IPVYQVNNL) was identified and submitted to IEDB (http://www.iedb.org/epId/91517)^49^ in 2009 by Kent J. Weinhold, Georgia D. Tomaras, Yongting Cai, Kelly Plonk, Scott Pruitt and Smita K. Nair.

### siRNA therapy

The most potential siRNAs against every gene are reported in this study. We have predicted the efficacy of exclusive siRNAs against all the genes of known ebolaviruses and found that none of the siRNAs is present against all the five ebolaviruses. There are 75 siRNAs, which are shared by any two species, and 1 siRNA is shared by three species (BDBV, EBOV and RESTV). In this study, we used a published siRNA efficacy predictor named ‘desiRm’, developed by our group. One may question the use of desiRm software when a large number of existing software packages for predicting the efficacy of siRNA are available. We used it because its standalone version allowed us to analyze a large number of 19mer oligonucleotides. Further, this algorithm has been shown to have comparable performance with the other methods^34^. Thus, selection is purely based on availability and not on any other criteria, like performance.

In this study, almost all the software used for predicting potential candidates for vaccine or siRNA therapeutics, were developed by our group. Some of these are extensively used software (e.g., ProPred, ProPred1 and CTLPred) developed in our laboratory. Some of the software packages are unique and not available from any other group, like IL4pred and IFNepitope. In conclusion, we have tried our best to use the most appropriate software packages to generate the above resources.

## Additional Information

**How to cite this article**: Dhanda, S. K. *et al.* A web-based resource for designing therapeutics against Ebola Virus. *Sci. Rep.* **6**, 24782; doi: 10.1038/srep24782 (2016).

## Supplementary Material

Supplementary Information

## Figures and Tables

**Table 1:** Distribution of 9mer peptides among five ebolaviruses.

Different types of proteins and peptides	Ebolavirus				
	BDBV	RESTV	SUDV	TAFV	EBOV
Proteins or antigens (including splice variants)	9	8	8	9	9
Number of 9mer peptides	5435	5129	5126	5427	5421
Unique 9mer peptides	4861	4841	4839	4853	4847
Exclusive 9mer peptides absent in humanreference proteomes	4861	4839	4838	4853	4847
Exclusive 9mer peptides absent in 1000Genomes-based proteomes	4861	4839	4838	4853	4846

**Table 2:** List of peptides conserved in different ebolaviruses excluding RESTV (non-pathogenic to human) and their immunogenicity.

Prediction ID	Immune status	Conserved in 4ebolaviruses	Conserved in 3ebolaviruses	Conserved in 2ebolaviruses	Present in 1ebolavirus
1	9mer peptides	812	1528	2835	14225
2	nHLApred	126	253	461	2028
3	MHC Class I	580	1083	2018	9758
4 (2 + 3)	CTL immunity	123	249	453	1989
5	MHC Class II	138	286	502	2380
**6 = (4 + 5)**	**T-cell immunity**	**21**	**43**	**77**	**310**
7	B-cell immunity	45	82	154	1020
**8 = (6 + 7)**	**Both immunity**	**0**	**2**	**3**	**14**
9	IFN-gamma	233	511	986	4744
10 = (5 + 9)	Th1 immunity	42	98	168	805
11	Interleukin-4	599	1141	2137	10553
12 = (5 + 11)	Th2 immunity	103	207	378	1762

**Table 3:** List of potential peptides or epitopes that can activate most of the arms of immune system.

Peptide sequence	Protein(ebolavirus)	CTLPredscore	# MHC-Ialleles	# MHC-IIalleles	# nHLApredalleles	B-epitopeprobability
ITAFLNIAL	VP30 (EBOV,TAFV, BDBV)	0.637	7	10	5	62.66
LTLCAVMTR	VP30 (EBOV,TAFV, BDBV)	0.54	7	9	12	60.05
LTRRGRLNR	L (EBOV, BDBV)	0.931	5	11	8	65.22
VLGYNPPNK	L (TAFV)	0.61	5	16	2	61.85
LARRGRLNR	L (TAFV)	0.624	4	9	8	61.98
FVEEWVIFR	L (SUDV)	0.755	9	9	3	83.95
LRMIEMDDL	L (EBOV)	0.55	7	9	5	65.01
LTRRGRMNR	L (EBOV)	0.527	5	10	5	67.78
VLGYSPPYR	L (SUDV)	0.456	5	9	1	63.34
WLADQKSRI	L (TAFV)	0.428	9	15	8	72.4
WRGRHRPKK	L (SUDV)	0.404	3	9	19	60.04
YVNLGFPSL	L (BDBV)	0.387	13	10	9	61.46
IPVYQVNNL	NP (EBOV)	0.982	24	10	3	64.65
MRHRRELQR	GP (SUDV)	0.465	3	9	3	65.99

**Table 4:** Number of predicted siRNAs (suppressors) against each target gene in different ebolaviruses.

Genes (variants)	Number of siRNA suppressors indifferent ebolaviruses				
	SUDV	RESTV	EBOV	TAFV	BDBV
NP	436	523	480	541	487
VP35	226	232	183	218	224
VP40	205	239	151	206	235
GP (Spike)	366	392	385	416	388
sGP (Soluble GP)	173	194	209	230	195
ssGP (Small Secreted GP)	–	–	145	184	157
VP30	232	234	226	217	237
VP24	195	164	198	194	163
L	1859	1995	2021	1901	1869
