# Supplementary material for: A web-based resource for designing therapeutics against Ebola Virus
Source: Sci Rep. 2016 Apr 26;6:24782. doi: 10.1038/srep24782 (PMC4845023; doi:10.1038/srep24782)

**A web-based resource for designing therapeutics against Ebola Virus**

Sandeep Kumar Dhanda^1^, Kumardeep Chaudhary^1^, Sudheer Gupta^1^, Samir Kumar Brahmachari^2^ and Gajendra P. S. Raghava^1^*

*Corresponding author

Gajendra P.S. Raghava

Chief Scientist and Head,

Bioinformatics Centre,

CSIR-Institute of Microbial Technology,

Sector 39A, Chandigarh,

India-160036

Email: [raghava@imtech.res.in](mailto:raghava@imtech.res.in)

^1^Bioinformatics Centre, CSIR-Institute of Microbial Technology,

Sector 39A, Chandigarh, India

^2^CSIR-Institute of Genomics and Integrative Biology,

Mathura Road, New Delhi, India.

**Supplementary information**

**Table Legends**

**Table S1**: Protein-wise conserved 9mer peptides among different ebolaviruses.

**Table S2:** Gene-wise conserved potential siRNAs among ebolaviruses taken in this study.

**Figure Legends**

**Figure S1:** Circos diagram for presentation of peptides in proteins of different ebolaviruses: A) SUDV, B) RESTV, C) EBOV, D) TAFV and E) BDBV.

**Figure S2:** Venn diagram to present the number of shared and exclusive peptides among

different ebolaviruses.

**Figure S3:** The Venn Diagram for protein wise-distribution of peptides and their sharing

among ebolaviruses: A) VP30, B) NP, C) L, D) VP24, E) VP35, F) VP40,

G) GP and H) sGP.

**Figure S4**: Mulitple sequence alignment of VP24 protein across all the ebolaviruses.

These alignments were generated using ClustalW and presented using Jalview software. All the alignment files can be found at <http://crdd.osdd.net/oscadd/ebola>/

**Figure S5:** Phylogenetic tree for VP24 protein of different ebolaviruses, generated based on

sequence similarity in different proteins. Rest of the alignment trees are available at <http://crdd.osdd.net/oscadd/ebola>/

**Figure S6:** Mulitple sequence alignment of VP24 mRNA across all the ebolaviruses.

These alignments were generated using ClustalW and presented using Jalview software. Rest of the alignment files are available from our web page at <http://crdd.osdd.net/oscadd/ebola>/

**Figure S7:** Venn diagram to present the number of shared and exclusive siRNA from

ebolaviruses

**Figure S8:** Circos diagram for presentation of siRNA of different ebolaviruses:

A) SUDV, B) RESTV, C) EBOV, D) TAFV and E) BDBV.

**Different splice variants of *GP* gene are represented as**

**GP=Spike Glycoprotein**

**sGP=Soluble Glycoprotein**

**ssGP=Small Soluble Glycoprotein**

**Table S1:** Protein-wise conserved 9mer peptides among different ebolaviruses.

| **S. No.** | **Protein** | **Symbol** | **Conserved 9mers** | **Conserved 9mer after excluding RESTV** |
| --- | --- | --- | --- | --- |
| 1 | Major Nucleoprotein | NP | 147 | 182 |
| 2 | Polymerase complex | VP35 | 35 | 63 |
| 3 | Matrix Protein | VP40 | 41 | 58 |
| 4 | Glycoprotein (Spike) | GP | 20 | 27 |
| 5 | Glycoprotein (Small Soluble) | ssGP | 9 | 9 |
| 6 | Minor Nucleoprotein | VP30 | 12 | 33 |
| 7 | Membrane Associated Protein | VP24 | 7 | 9 |
| 8 | Polymerase | L | 358 | 431 |

**Table S2:** Gene-wise conserved potential siRNAs among ebolaviruses taken in this study.

| **Gene** | **siRNAs common in any 2 ebolaviruses (including RESTV)** | **siRNAs common in any ebolaviruses (excluding RESTV)** |
| --- | --- | --- |
| **NP** | 11 | 7 |
| **VP35** | 1 | 0 |
| **VP40** | 1 | 1 |
| **GP** | 26 | 14 |
| **sGP** | 18 | 12 |
| **ssGP** | 8 | 8 |
| **VP30** | 5 | 3 |
| **VP24** | 3 | 1 |
| **L** | 18 | 12 |

**Figure S1: Circos diagram for presentation of peptides in proteins of different ebolaviruses: A) SUDV, B) RESTV, C) EBOV, D) TAFV and E) BDBV.**


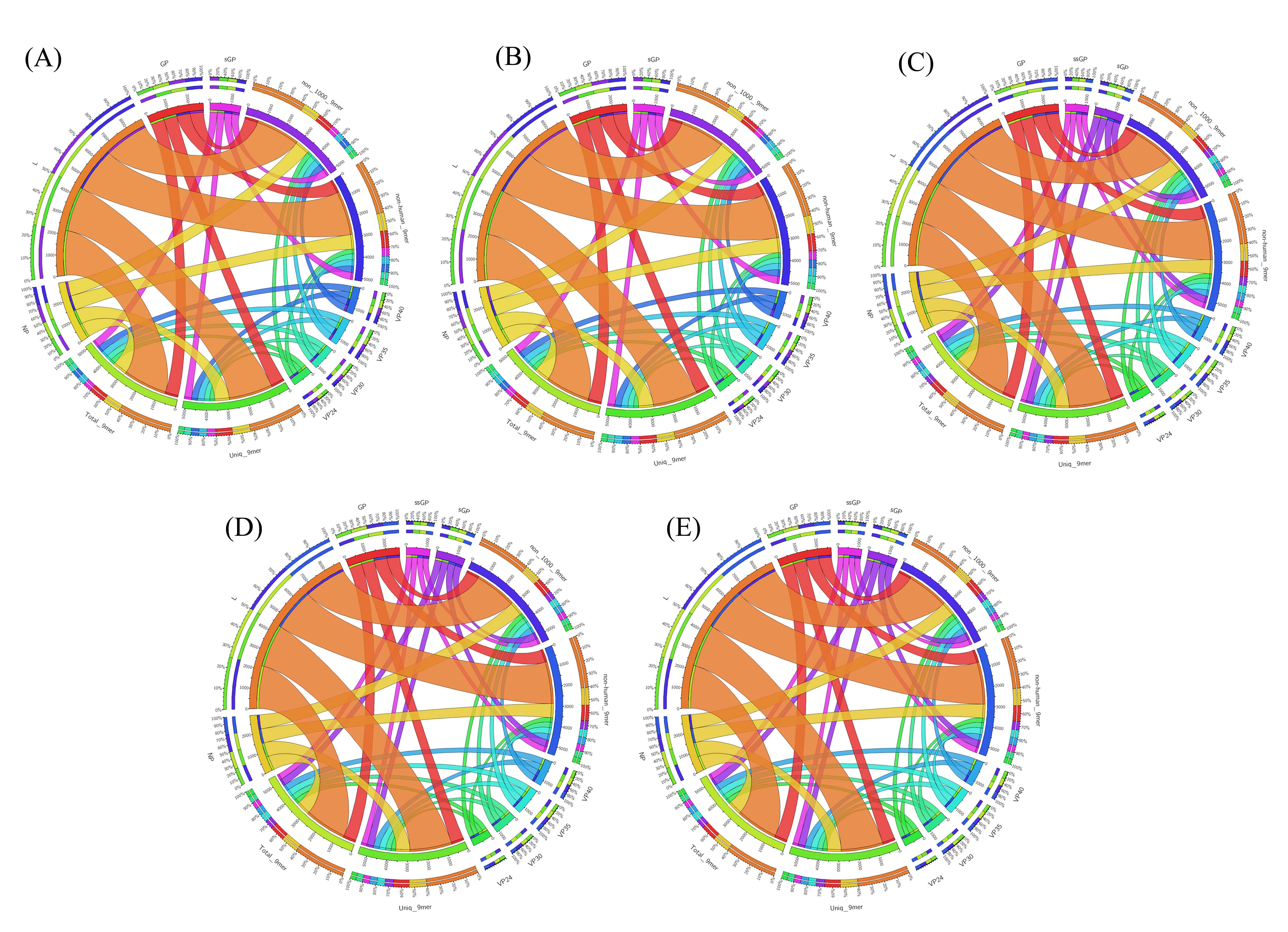


**Figure S2: Venn diagram to present the number of shared and exclusive peptides among different ebolaviruses.**

**

**

**Figure S3: The Venn Diagram for protein wise-distribution of peptides and their sharing**

**among different ebolaviruses: A) VP30, B) NP, C) L, D) VP24, E) VP35, F) VP40,**

**G) GP and H) sGP.**


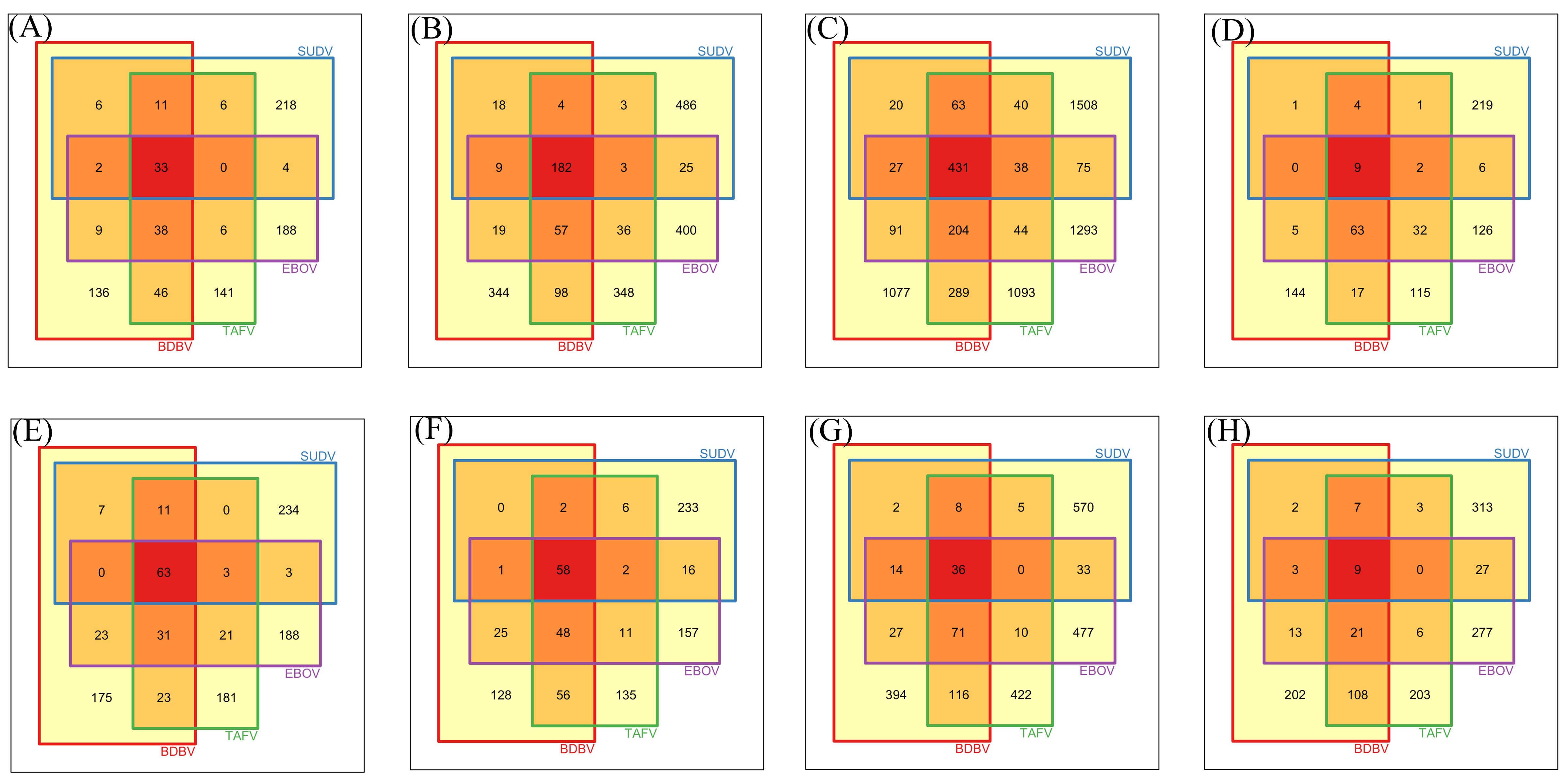


**Figure S4: Mulitple sequence alignment of VP24 protein across all the ebolaviruses. These alignments were generated using ClustalW and presented using Jalview software. All the alignment files can be found at** [**http://crdd.osdd.net/oscadd/ebola**](http://crdd.osdd.net/oscadd/ebola)**/**


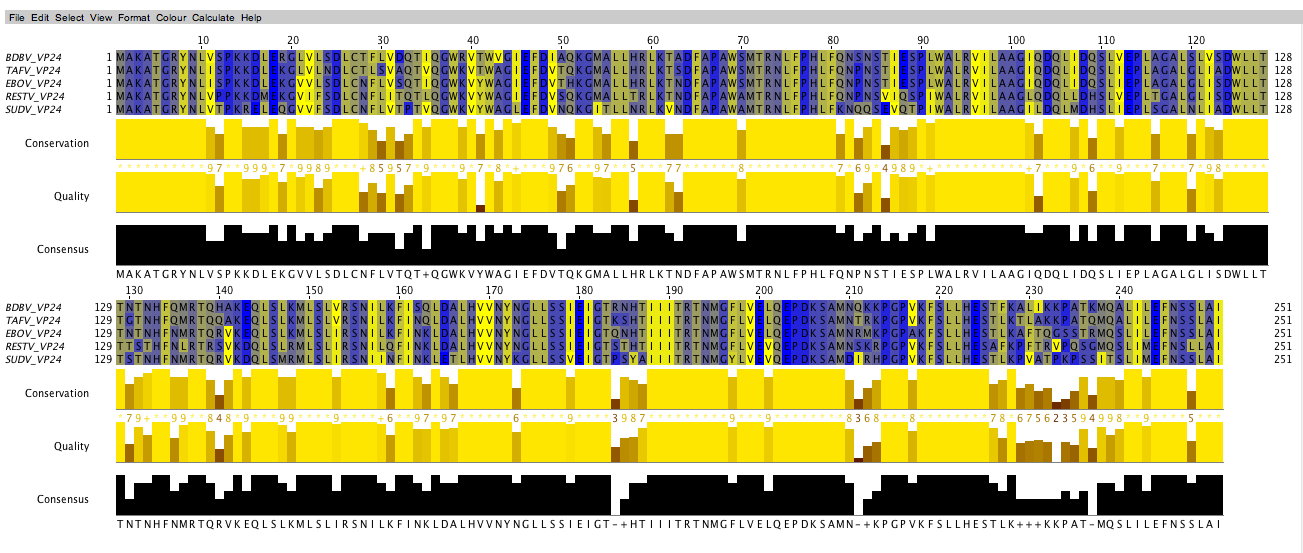


**Figure S5: Phylogenetic tree for VP24 protein of different ebolaviruses, generated based on sequence similarity in different proteins. Rest of the alignment trees are available at** **http:/crdd.osdd.net/oscadd/ebola/**

**
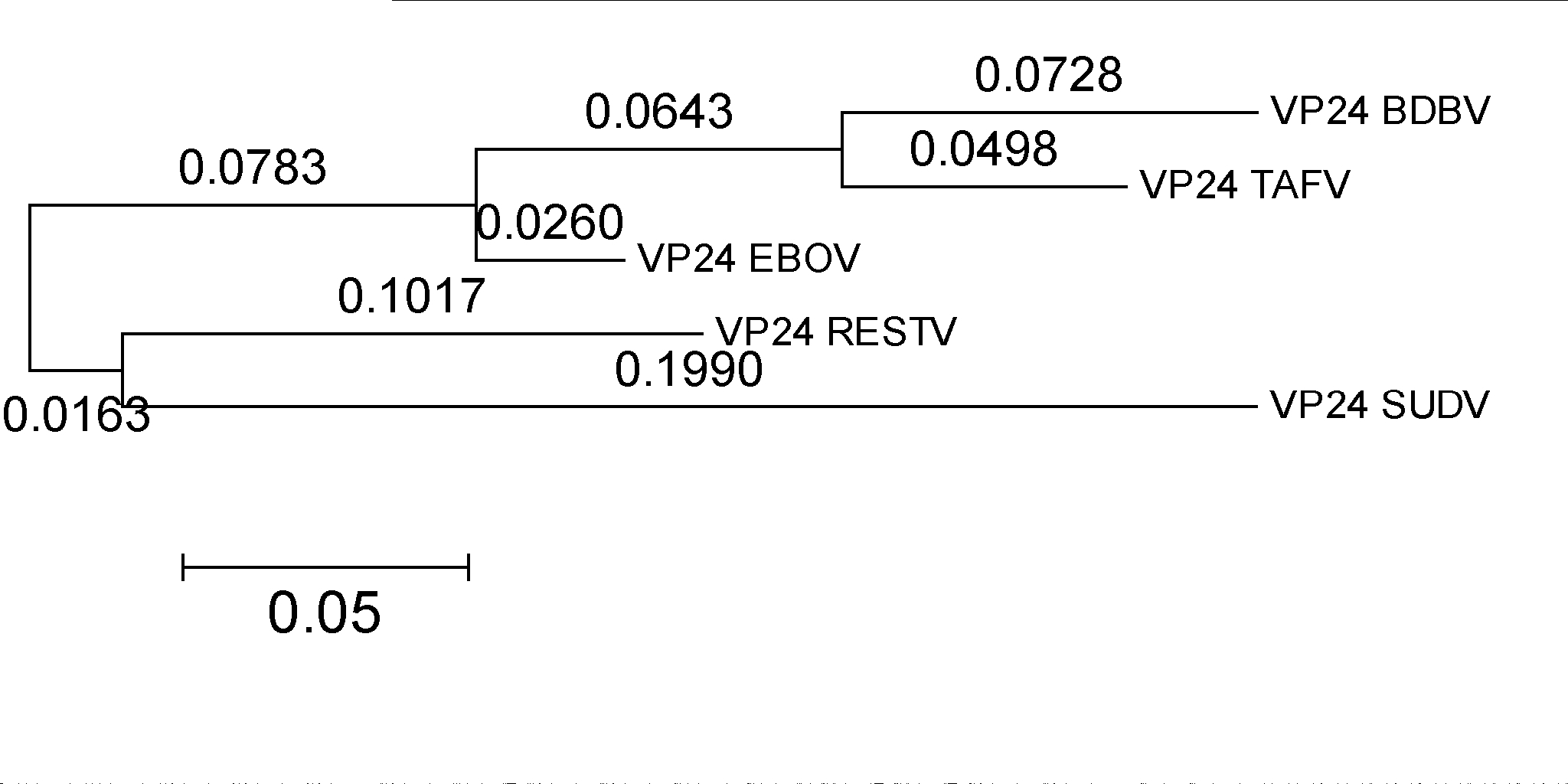
**

**Figure S6: Mulitple sequence alignment of VP24 mRNA across all the ebolaviruses. These alignments were generated using ClustalW and presented using Jalview software. Rest of the alignment files are available from our web page at** [**http://crdd.osdd.net/oscadd/ebola**](http://crdd.osdd.net/oscadd/ebola)**/**


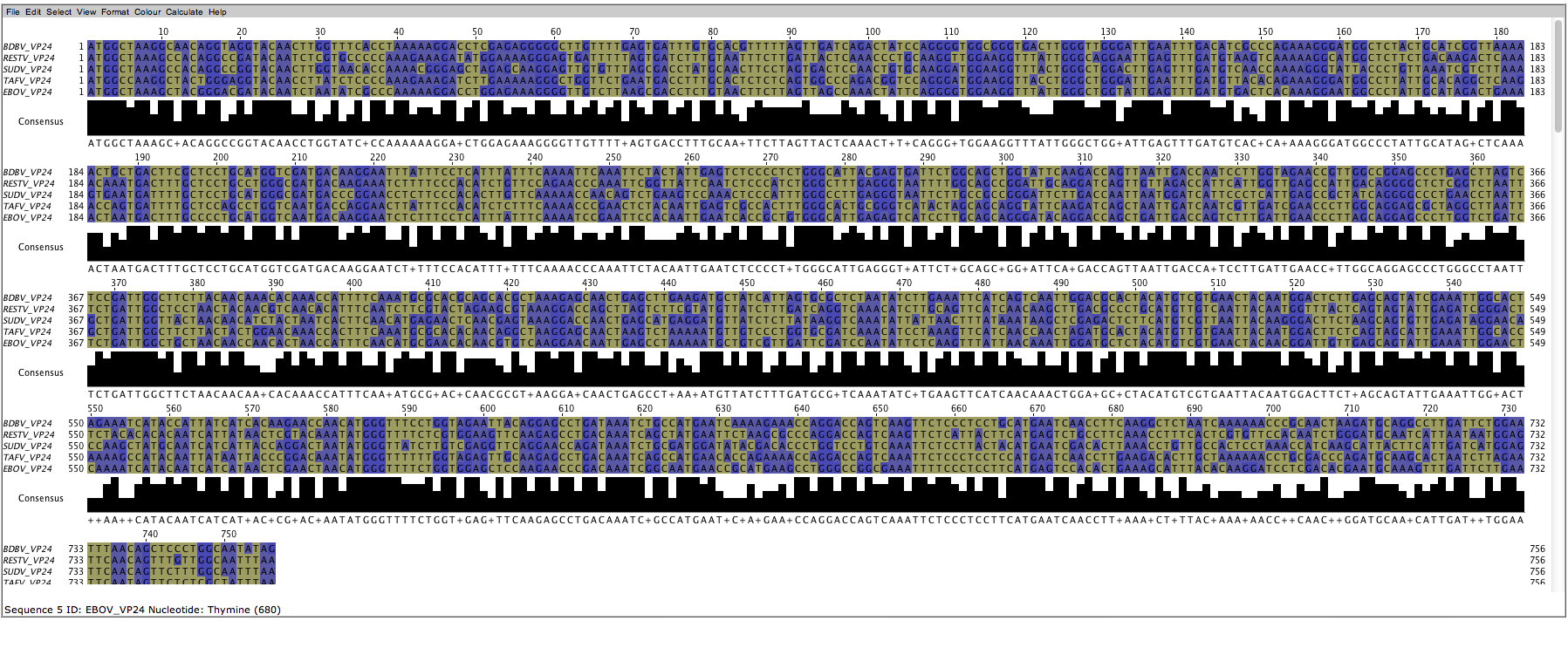


**Figure S7: Venn diagram to present the number of shared and exclusive siRNA from five ebolaviruses.**


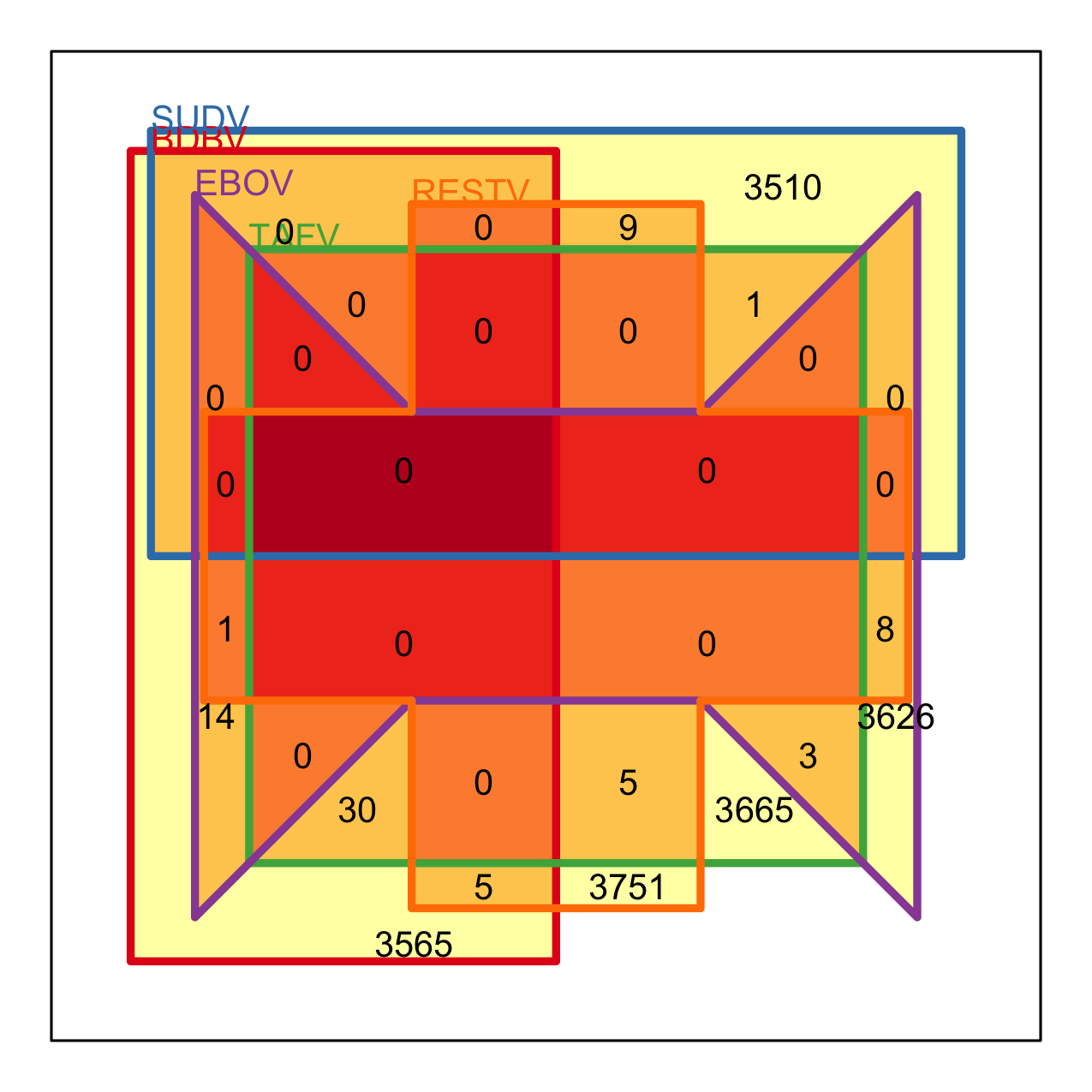


**Figure S8: Circos diagram for presentation of siRNA of different ebolaviruses:**

1. **SUDV, B) RESTV, C) EBOV, D) TAFV and E) BDBV.**


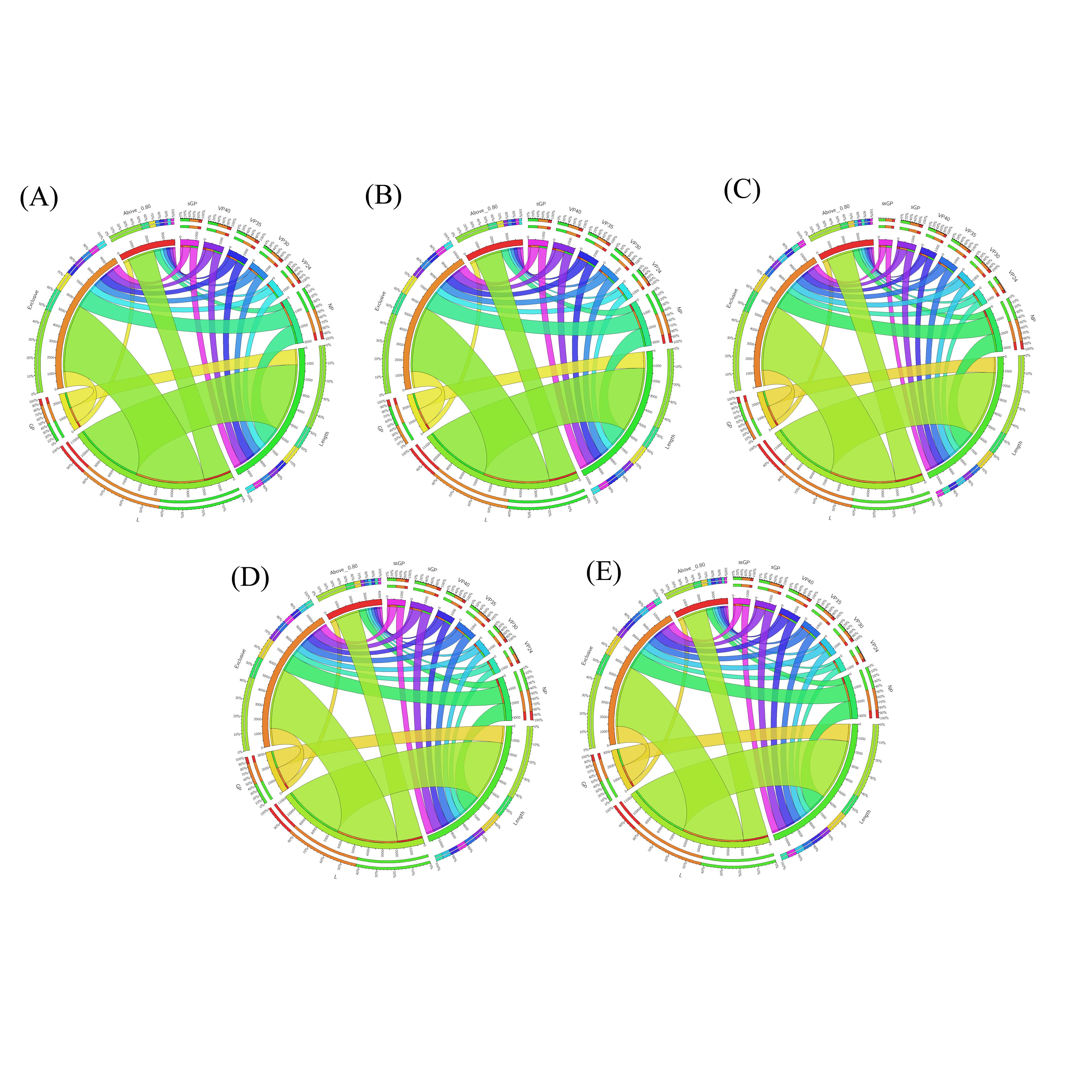

Supplement: Supplementary Information [file srep24782-s1.doc]
